# CD30^+^ extranodal natural killer/T-cell lymphoma mimicking phlegmonous myositis: A case report

**DOI:** 10.3892/ol.2014.1924

**Published:** 2014-02-28

**Authors:** YAN-JIA YANG, YA-XIN LI, YAN-BIN LIU, MEI YANG, KAI LIU

**Affiliations:** Center of Infectious Disease, West China Hospital, Sichuan University, Chengdu, Sichuan 610041, P.R. China

**Keywords:** extranodal natural killer/T-cell lymphoma, nasal-type, phlegmonous myositis, fever of undetermined origin

## Abstract

The current study presents a case of a 23-year-old male with CD30^+^ nasal-type extranodal natural killer/T-cell lymphoma (NKTL), with unusual clinical features mimicking phlegmonous myositis. The patient initially presented with swelling and tenderness of the left lower limb, particularly around the left ankle. One month later, pharyngalgia and fever developed and the patient was treated with antibiotics for the phlegmonous inflammation, however, the symptoms were not relieved. A muscle biopsy was performed on the lesion and revealed diffuse infiltration of atypical lymphoid cells with irregular nuclei. Immunohistochemistry showed staining for CD3ɛ(−), CD20(−), CD45(+), CD30(+) and CD56(+) presented with positive staining for certain tumor cells, granzyme B(+), activin receptor-like kinase 1(−), Ki-67(+) and Epstein-Barr virus-encoded small RNA(+), which indicated nasal-type extranodal NKTL. The present case emphasized that extranodal NKTL may be a rare cause of phlegmonous inflammation and fever of undetermined origin.

## Introduction

Nasal-type extranodal natural killer/T-cell lymphoma (NKTL) is a rare type of lymphoma, which is associated with the Epstein-Barr virus (EBV). NKTL has also been referred to as lethal midline granuloma and polymorphic reticulosis. When NKTL occurs outside the nasal cavity, such as in the skin, soft tissue, gastrointestinal tract and other extranasal sites, it may have various presentations (1–7). As a result of advances in immunohistochemistry, the disease can now be more easily identified. The current study presents a case of a 23-year-old male with CD30^+^ nasal-type extranodal NKTL, with skeletal muscle involvement mimicking phlegmonous myositis and fever of undetermined origin (FUO). In addition, the diagnosis of extranodal NKTL and FUO is discussed. The patient provided written informed consent.

## Case report

### Patient history

In August 2011, a 23-year-old male presented with swelling, tenderness and high skin temperature of the left lower limb, particularly around the ankle as the patient did not have a fever the patient did not seek treatment. However, one month later, pharyngalgia and fever developed and the patient was treated with antibiotics for the inflammation at a local health center, however, the symptoms were not improved. The patient was subsequently transferred to the Chengdu First People’s Hospital (Chengdu, China) for hospitalization.

### Laboratory tests

The laboratory tests (9 Aug, 2011) showed leukopenia, with a white blood cell (WBC) count of 1.45×10^9^/l and 0% eosinophils. The serum biochemistry revealed alanine aminotransferase (ALT) levels of 114 IU/l (normal range, <55 IU/l), aspartate transaminase (AST) levels of 83 IU/l (normal range, <46 IU/l), lactate dehydrogenase (LDH) levels of 479 IU/l (normal range, 110–220 IU/l) and creatine phosphokinase levels of 736 IU/l (normal range, 25–190 IU/l). B type ultrasound (9 Aug, 2011) showed splenomegaly and swelling of the soft tissue in the left lower limb, with reduced blood flow and a small mass in the vein. The bone marrow biopsy was normal. A complete blood count (CBC) was performed (12 Aug, 2011) and showed a WBC count of 10.79×10^9^/l (84.6% neutrophils).

### Treatment and further testing

Multiple antibiotics were administered continuously, however, the patient showed no clinical response. During hospitalization, the patient’s condition deteriorated, with the highest temperature reaching 40.4°C and weight loss of 5 kg over two months. The patient was referred to the Infection Disease Center (West China Hospital, Chengdu, China) due to FUO. On physical examination, purulent secretion was found in the patient’s nose and the spleen was palpable 5 cm below the costal margin. The laboratory tests (22 Aug, 2011) showed a WBC count of 1.57×10^9^/l (36.5% neutrophils), platelet count of 71×10^9^/l, ALT levels of 184 IU/l, AST levels of 197 IU/l, LDH levels of 554 IU/l and γ-glutamyltransferase levels of 432 IU/l.

### Computed tomography, magnetic resonance imaging and biopsy

**Figure 1:**
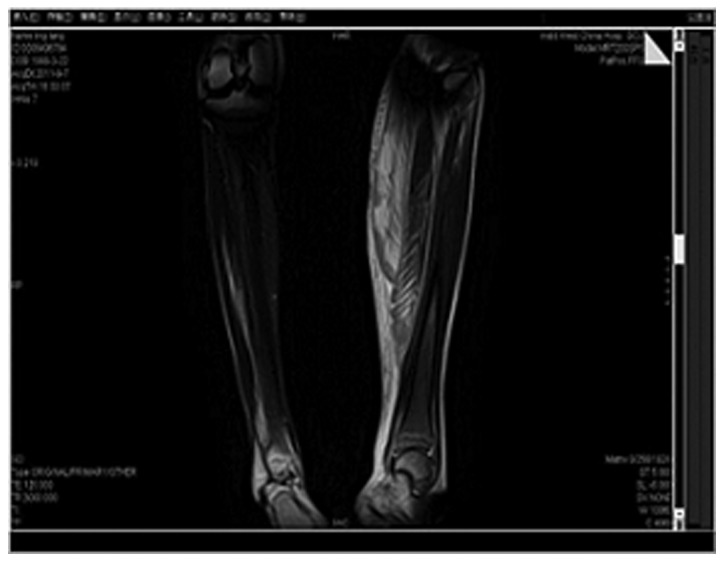
Diffuse infiltrative lesion was hyperintense on T1-weighted image and showed enhancement.

A computed tomography scan of the laryngopharynx identified inflammatory changes, particularly in the left nasopharynx and the soft palate, as well as hyperplasia of the submandibular lymph nodes. Magnetic resonance imaging of the lower extremity detected a diffuse infiltrative lesion, mimicking phlegmonous myositis (Fig. 1). A bone marrow biopsy was performed again and showed a depression with normal morphology. Prominent ulceration and necrosis with fungal elements (usually oidiomycetic) were observed in the soft palate by pharyngoscopy. Taking into consideration the fever, ulceration in the nasopharynx, splenomegaly, weight loss and cachexy, the diagnosis of extranodal NKTL was determined. In addition, the muscle biopsy of the left limb and repeat biopsy of the pharynx was of concern. Diffuse infiltration of medium-sized atypical lymphoid cells with irregular nuclei was shown in the gastrocnemius biopsy.

### Immunohistochemical analysis

**Figure 2:**
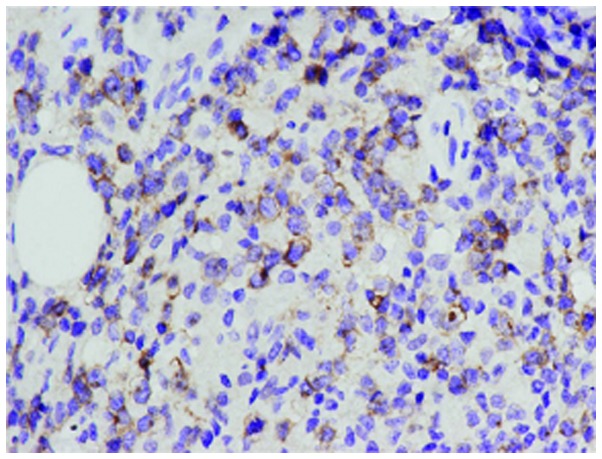
Immunohistochemistry of the tumor cells showed multifocal positive staining for CD30 (3,3′-diaminobenzidine staining; magnification, ×500).

**Figure 3:**
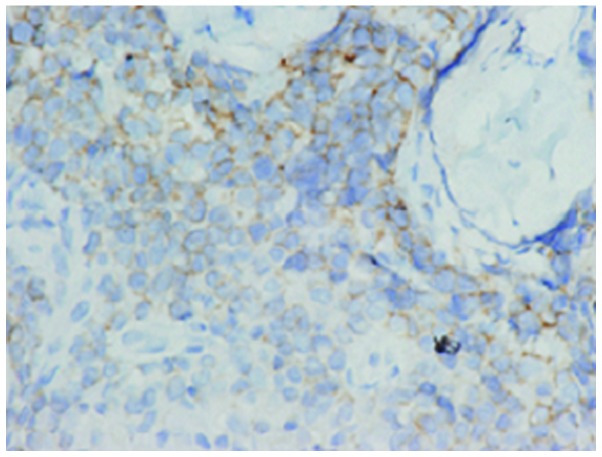
Immunohistochemistry revealed that the majority of tumor cells were positive CD56 staining in portions (3,3′-diaminobenzidine staining magnification, ×500).

Immunohistochemical staining [avidin-biotin-peroxidase and elivision methods (8)] was performed on the paraffin-embedded sections using the following commercial antibodies: rabbit monoclonal anti-CD3 (SP7, 1:50; LabVision-NeoMarkers, Fremont, CA, USA), polyclonal rabbit anti human CD3ɛ (1:50; Dakopatts, Glostrup, Denmark), monoclonal mouse anti-CD20 (L26, 1:100; Zymed, South San Francisco, CA, USA), monoclonal mouse anti-CD30 (Ber-H2, 1:50; LabVision-NeoMarkers), monoclonal mouse anti-CD45RO (UCHL1, 1:100; Zymed), monoclonal mouse anti-CD56 (123C3, 1:100; Zymed), monoclonal mouse anti-GranzymeB (GZB01, 1:100; LabVision-NeoMarkers), polyclonal rabbit anti-Ki67 (MIB-1, 1:100; LabVision- NeoMarkers), monoclonal mouse anti-human ALK1 (ALK1, 1:100; Dako, Carpinteria, CA, USA). *In situ* hybridization for EBV to encode RNA (EBER1/2) was performed on fixed paraffin-embedded sections. The fluorescein isothiocynate-conjugated EBER peptide nucleic acid probe (Y5200) was purchased from Dakopatts. The histochemical staining was positive for CD45, CD30 (Fig. 2), the majority of CD56 cells (Fig. 3), granzyme B and Ki-67, and negative for CD3ɛ, CD20 and activin receptor-like kinase 1 (ALK1). The *in situ* hybridization for EBV-encoded small RNA (EBER) was strongly positive.

### Follow-up

Based on the clinical manifestations and pathological observations, a diagnosis of nasal-type extranodal NKTL was determined and the patient was transferred to the Department of Hematology (West China Hospital, Chengdu, China) for chemotherapy with GL-IDE: Gemcitabine (1.6 g on days 1 and 8); L-asparaginase (10,000 U/m^2^ on days 4, 6, 8 and 10); Ifosfamide (1.6 g on days 1, 2 and 3); Dexamethasone (20 mg on days 1, 2, 3 and 4); Etoposide (160 mg on days 1, 2 and 3). The patient succumbed as a result of the rapid progression of the disease two months later.

## Discussion

Nasal-type extranodal NKTL has also been referred to as lethal midline granuloma or polymorphic reticulosis and may have variable presentations depending on the predominant site of involvement. To date, only sporadic cases concerning skin, muscle and ocular involvement have previously been reported (1–4). Min *et al* (5) reported a case of extranodal NKTL with muscle involvement that initially manifested with granulomatous myositis, with a normal CBC count and without fever. In addition, Paik *et al* (6) reported a case of extranodal NKTL with skeletal muscle involvement and heavy eosinophilic infiltration, with a peripheral blood eosinophilia of 22.2% on CBC count. However, the current patient presented with swelling, tenderness and a high skin temperature of the left lower limb, followed by hyperpyrexia. The patient’s WBC count reached 10.79×10^9^/l (84.6% neutrophils and 0% eosinophils), which mimicked phlegmonous myositis. The present case illustrated the requirement for caution when diagnosing NKTL as it may be a rare cause of unexplained phlegmonous myositis, particularly when accompanied with FUO and symptoms in the nasopharynx.

Extranodal NKTL is characterized by vascular damage, prominent necrosis and is associated with the EBV (7,9–11). The typical immunophenotype for extranodal NKTL is CD2^+^, CD56^+^, surface CD3^−^ and cytoplasmic CD3ɛ^+^, as well as positivity for cytotoxic molecules (granzyme B, T-cell intracellular antigen 1 and perforin) (13–15). As observed in the present case, early biopsy may give a false indication of an inflammatory disorder due to the paucity of neoplastic cells; however, in NKTL, a large number of inflammatory cells are recruited by the innate natural killer cell immune response and extensive necrosis is caused by angiodestructive tumor cells. However, in the present study, the repeat biopsy (which showed atypical lymphoid cells positive for granzyme B, EBER, CD45 and CD56, and negative for CD20 and ALK1) showed features that were consistent with NKTL. This emphasized the requirement for a repeat biopsy when NKTL is suspected, as necrosis is often present in biopsy. The biological meaning for the positivity of CD30 is not clearly understood. As CD30 is normally associated with anaplastic large cell lymphoma, the positivity for CD30 has been reported in few cases of cutaneous NKTL. The present case showed that extranodal NKTL must be considered as a differential diagnosis of CD30^+^ and CD56^+^ lymphoma.

In conclusion, in patients who exhibit FUO, infectious diseases, malignancies, collagen vascular diseases and a variety of miscellaneous disorders must be considered. The cause of FUO is partially age-related and neoplastic disorders have replaced infectious diseases as the most common cause of FUO; non-Hodgkin’s lymphoma is the first cause of FUO that has been linked to cancer (16,17). Although neoplastic disorders are more commonly observed in elderly patients, the present patient was 23-years-old, which is relatively different from the median age (50 years) of those with NKTL, indicating that NKTL is occasionally found in younger patients.

## References

[1] Cimino L, Chan CC, Shen D, Masini L, Ilariucci F, Masetti M (2009). Ocular involvement in nasal natural killer T-cell lymphoma. Int Ophthalmol.

[2] Chen CS, Miller NR, Lane A, Eberhart C (2008). Third cranial nerve palsy caused by intracranial extension of a sino-orbital natural killer T-cell lymphoma. J Neuroophthalmol.

[3] Yousuf SJ, Kumar N, Kidwell ED, Copeland RA (2011). Rapidly fatal nasal natural killer/T-cell lymphoma: orbital and ocular adnexal presentations. Orbit.

[4] Stokkermans-Dubois J, Jouary T, Vergier B, Delaunay MM, Taieb A (2006). A case of primary cutaneous nasal type NK/T-cell lymphoma and review of the literature. Dermatology.

[5] Min HS, Hyun CL, Paik JH, Jeon YK, Choi G, Park SH (2006). An autopsy case of aggressive CD30^+^ extra-nodal NK/T-cell lymphoma initially manifested with granulomatous myositis. Leuk Lymphoma.

[6] Paik JH, Jeon YK, Go HJ, Kim CW (2011). Extranodal NK/T cell lymphoma accompanied by heavy eosinophilic infiltration and peripheral blood eosinophilia, involving skeletal muscles. Korean J Pathol.

[7] Wood PB, Parikh SR, Krause JR (2011). Extranodal NK/T-cell lymphoma, nasal type. Proc (Bayl Univ Med Cent).

[8] He Q, Xu H, Shao MM, Yu XW (2007). Evaluation of rapid immunohistochemical staining technique in intraoperative frozen section diagnosis of breast tumors. Chinese J Clin Exp Pathol.

[9] Salinas F, Mulero F, Marin M, Padilla A, Fernandez I (2004). Fever of unknown origin (FUO), scintigraphy with Gallium 67 citrate scintigraphy and malignant lymphoma of soft tissues. Rev Esp Med Nucl.

[10] Li YX, Fang H, Liu QF, Lu J, Qi SN, Wang H (2008). Clinical features and treatment outcome of nasal-type NK/T-cell lymphoma of Waldeyer ring. Blood.

[11] Li YX, Liu QF, Fang H, Qi SN, Wang H, Wang WH (2009). Variable clinical presentations of nasal and Waldeyer ring natural killer/T-cell lymphoma. Clin Cancer Res.

[12] Qin W, Yin Z, Madge SN (2009). Acute presentation of nasal-type natural killer/T-cell lymphoma of the orbit. Eur J Ophthalmol.

[13] Yang Y, Luo Q, He W, Tang L (2007). Primary ocular natural killer/T-cell lymphomas: clinicopathologic features and diagnosis. Ophthalmologica.

[14] Takahashi E, Ohshima K, Kimura H, Hara K, Suzuki R, Kawa K (2011). Clinicopathological analysis of the age-related differences in patients with Epstein-Barr virus (EBV)-associated extranasal natural killer (NK)/T-cell lymphoma with reference to the relationship with aggressive NK cell leukaemia and chronic active EBV infection-associated lymphoproliferative disorders. Histopathology.

[15] Kim JE, Kim YA, Jeon YK, Park SS, Heo DS, Kim CW (2003). Comparative analysis of NK/T-cell lymphoma and peripheral T-cell lymphoma in Korea: Clinicopathological correlations and analysis of EBV strain type and 30-bp deletion variant LMP1. Pathol Int.

[16] Ferrari P, Schneemann M, Zimmerli L (2009). FUO: fever of unknown origin. Praxis (Bern 1994).

[17] Arce-Salinas CA, Morales-Velázquez JL, Villaseñor-Ovies P, Muro-Cruz D (2005). Classical fever of unknown origin (FUO): current causes in Mexico. Rev Invest Clin.

